# Importance of stress-response genes to the survival of airborne *Escherichia coli* under different levels of relative humidity

**DOI:** 10.1186/s13568-017-0376-3

**Published:** 2017-03-24

**Authors:** Tsz Wai Ng, Wing Lam Chan, Ka Man Lai

**Affiliations:** Department of Biology, Hong Kong Baptist University, Kowloon Tong, Hong Kong, SAR

**Keywords:** Bioaerosols, Airborne bacteria, Relative humidity, Stress response

## Abstract

Other than the needs for infection control to investigate the survival and inactivation of airborne bacterial pathogens, there has been a growing interest in exploring bacterial communities in the air and the effect of environmental variables on them. However, the innate biological mechanism influencing the bacterial viability is still unclear. In this study, a mutant-based approach, using *Escherichia coli* as a model, was used to prove the concept that common stress-response genes are important for airborne survival of bacteria. Mutants with a single gene knockout that are known to respond to general stress (*rpoS*) and oxidative stress (*oxyR*, *soxR*) were selected in the study. Low relative humidity (RH), 30–40% was more detrimental to the bacteria than high RH, >90%. The log reduction of ∆*rpoS* was always higher than that of the parental strain at all RH levels but the ∆*oxyR* had a higher log reduction than the parental strain at intermediate RH only. ∆*soxR* had the same viability compared to the parental strain at all RH levels. The results hint that although different types and levels of stress are produced under different RH conditions, stress-response genes always play a role in the bacterial viability. This study is the first reporting the association between stress-response genes and viability of airborne bacteria.

**Electronic supplementary material:**

The online version of this article (doi:10.1186/s13568-017-0376-3) contains supplementary material, which is available to authorized users.

## Introduction

Other than the needs for infection control to investigate the survival and inactivation of airborne bacterial pathogens, there has been a growing interest in exploring bacterial communities in the air and the effect of environmental variables on them (Franzetti et al. 2011; Tang 2009; Mohr 2007; Sun and Ariya 2006). Various bioaerosol studies have been conducted to determine the survival rate of airborne bacteria under different conditions to explain the bacterial diversity, predict the risk of airborne disease transmission and identify appropriate infection-control strategies (Parienta et al. 2011; Thompson et al. 2011; Cox 1986). However, very little knowledge has been accumulated regarding the innate biological mechanism influencing the bacterial viability. Stress response is one of the major mechanisms to help bacteria to overcome harsh environmental conditions. The genetic response and regulation of bacteria subjected to different types of environmental stress, such as oxidative stress, dehydration and cold stress, have been explored in many media such as water, soil and food, but never in an airborne context (Cabiscol et al. 2010; Chung et al. 2006). Understanding the stress response mechanism could provide a new biotechnology and engineering target to predict and control infection risks and facilitate the application of bioaerosol techniques to other fields [e.g. cloud condensation and climate change (Sun and Ariya 2006)]. As reported in some previous studies (Krumins et al. 2014; Dimmick et al. 1975), metabolic activities have been detected in bacterial aerosols. We hypothesize that stress-response genes also play a role to help the survival of airborne bacteria as in other environmental media.

The aim of this study is to examine some common stress-response genes in *Escherichia coli* to test this hypothesis. We selected several mutants, each with a single gene knockout. These genes are known to respond to general stress (*rpoS*) and oxidative stress (*oxyR, soxR*), in ways that may be relevant to bioaerosol survival, according to our literature review (Parienta et al. 2011; Tang 2009; Mohr 2007; Cox and Baldwin 1967). By comparing the extent of the log reduction in bacterial survival between the mutant and parental strain, we can identify the genes that are associated with the airborne viability. Relative humidity (RH) is the most widely studied environmental factor to affect bioaerosol evaporation and survival (Parienta et al. 2011; Dunklin and Puck 1948) and was investigated in this study.

## Materials and methods

### Bacterial strains

*Escherichia coli* was selected as a model bacterium due to its extensive use in bioaerosol and other stress response studies. *E. coli* BW25113 (the parental strain) and its isogenic deletion mutants were purchased from Coli Genetic Stock Center (CGSC, Yale University, USA) (Baba et al. 2006) (Table 1).

**Table 1:** The genetic information of the bacteria used in the present study

Strain name	GCSC number	Deleted gene	Mutation function
BW25113	7636	None	Not applicable
JW5437-1	11,387	*rpoS*	Master regulator of the general stress response in *E. coli*. In addition, *rpoS* transcribes a significant fraction of genes related to sugar and polyamine metabolism in response to cellular stresses and in nucleic acid synthesis and modification (Eisenstark et al. 1996)
JW3933-3	12,039	*oxyR*	“Oxidative stress regulator,” is the transcriptional dual regulator for the expression of antioxidant genes in response to oxidative stress, in particular, elevated levels of hydrogen peroxide (Kullik et al. 1995)
JW4024-1	10,892	*soxR*	“Superoxide response protein,” is negatively autoregulated and controls the transcription of the regulon involved in defense against redox-cycling drugs (Demple 1996)

### Bacterial culture

Fresh cultures of *E. coli* and its mutants were grown in Luria–Bertani medium (Affymetrix Inc., USA) at 37 °C to stationary phase for 16 h with constant shaking at 150 rpm. Stationary phase was determined by using growth curves. Our own and previous studies showed that exponential phase bacteria died significantly during airborne suspension so they could not be examined in this study. Next, the bacterial cells were harvested by centrifugation at 3000×*g* for 7 min, then washed and re-suspended in phosphate buffer saline (PBS, pH 7.4) and transferred to a six-jet Collison nebulizer (BGI Inc., USA) for nebulization at 20 psi. The reason of suspending and aerosolizing the bacteria in PBS rather than directly from the culture media is to minimize the variation and unknown composition of the culture media. It is a general procedure applied in bioaerosol studies.

### Survival during airborne suspension

To test the bioaerosol survival, each bacterial suspension was nebulized for 3 min (N_0_) in the nebulizer at room temperature, 20 ± 2 °C, and the aerosols generated were suspended in a cylindrical chamber (diameter × height: 50.8 cm × 58 cm, volume: 87 L). The air temperature in the chamber was the same as the room temperature, and RH was adjusted by either spraying sterile water or purging dehumidifying air into the chamber to achieve low (30–40%), intermediate (40–60%) and high (>90%) levels of RH. The temperature and RH of the chamber were measured by a digital hygrometer. After 30 min of airborne suspension, the bacterial cells were sampled on a 0.22 μm mixed cellulose ester filter (Advantec, Japan) at flow rate of 28 L/min for 3 min (N_30_). Immediately after sampling, the filter was placed into 5 mL of PBS and vigorously shaken in a vortex for 30 s to elute the deposited bacteria. Both culturable and DNA counts of the collected bacteria were analyzed to determine the final bacterial concentration. The culturability of the bacterial cells was determined by the plate-count method (spreading the sample on tryptone soy agar (TSA) and incubated at 37 °C for 24 h) and the DNA counts by quantitative polymerase chain reaction (qPCR).

Bacterial DNA was extracted using a QIAamp DNA Mini Kit (Qiagen, Germany) following the manufacturer’s protocol. The concentration of the extracted DNA samples was determined by qPCR using a QuantiNova™ SYBR^®^ Green PCR Kit (Qiagen, Germany) with the forward primer 784 (5′-GTG TGA TAT CTA CCC GCT TCG C-3′) and the reverse primer 866 (5′-AGA ACG GTT TGT GGT TAA TCA GGA-3′). These primers bind to the *uidA* gene, which is specific to *E. coli* and thus used in *E. coli* determination (Fram and Obst 2003). The thermocycling program of the AB StepOne RT-PCR System (AB, USA) consisted of an initial activation cycle at 95 °C for 2 min, followed by 40 cycles of denaturation at 95 °C for 5 s and combined annealing/extension at 60 °C for 10 s. The *E. coli* BW25113 culture was used to set a standard calibration curve. To account for the potential loss of bioaerosols during aerosolization and sampling, a normalized survival ratio (N) was calculated as shown in Eq. 1. Reagents and buffers used in the study were autoclaved to eliminate DNase contamination.

1 $${\text{N}} = \frac{{{\text{number of culturable bacterial cells }}({\text{plate-count data}})}}{{{\text{total number of bacterial cells }}({\text{qPCR data}})}}$$

To assess the change in the airborne bacterial survival, the log reduction between the normalized culturable bacterial count before (N_0_) and after 30 min (N_30_) of aerosolization was calculated as shown in Eq. 2.

2 $${\text{Log}}\,{\text{reduction}} = Log_{10} N_{0} - log_{10} N_{30}$$

### Effect of nebulization and filter sampling on the bacterial survival

The survival percentage of the bacteria was determined before and after nebulization by using plate-counting method to prove that nebulization did not inactivate the bacteria. For filter sampling, two experiments were conducted to support that air filtration did not conceal the effect of airborne suspension on the viability of the bacteria. The details of the methods and the results were described in the Additional file 1: Figures S1, S2 and S3.

### Statistical analysis

The log reduction in bacterial survival under each RH condition was compared using one-way analysis of variance (ANOVA) with Duncan’s post hoc test (SPSS v. 23) in order to determine whether a particular gene deletion makes a difference in the bacterial survival as compared to the parental strain aerosolized under the same condition. The difference between means with a *p* value lower than 0.05 (*p* < 0.05) was regarded as statistically significant. The same analysis was also conducted by grouping all mutants at different RH conditions together in one model (Additional file 1: Figures S4, S5 and S6). This analysis showed the overall mutant comparison.

## Results

**Fig. 1:**
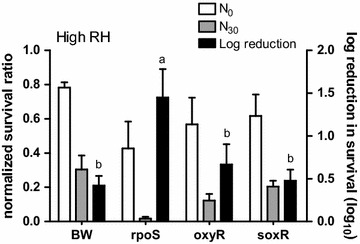
The normalized survival ratio and log reduction in survival of the bacteria at high RH. The normalized survival ratio before (N_0_) and after 30 min (N_30_) of aerosolization (*left Y-axis*) and log reduction in survival (*right Y-axis*) of the parental strain (BW), ∆*rpoS,* ∆*oxyR* and *∆soxR*. Temperature: 20 ± 2 °C. *Error bars* represent the standard deviation of replicates (n = 3). The log reduction of different mutants was statistically analyzed by one-way ANOVA. Grouping was conducted with post-hos test Duncan analysis, and the *letters* above the *bars* represent different grouping

**Fig. 2:**
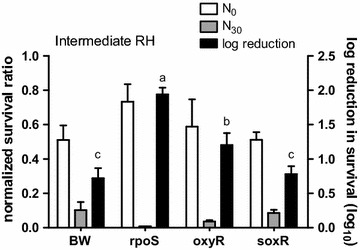
The normalized survival ratio and log reduction in survival of the bacteria at intermediate RH. The normalized survival ratio before (N_0_) and after 30 min (N_30_) of aerosolization (*left Y-axis*) and log reduction in survival (*right Y-axis*) of the parental strain (BW), ∆*rpoS,* ∆*oxyR* and *∆soxR*. Temperature: 20 ± 2 °C. *Error bars* represent the standard deviation of replicates (n = 3). The log reduction of different mutants was statistically analyzed by one-way ANOVA. Grouping was conducted with post-hos test Duncan analysis, and the *letters* above the *bars* represent different grouping

**Fig. 3:**
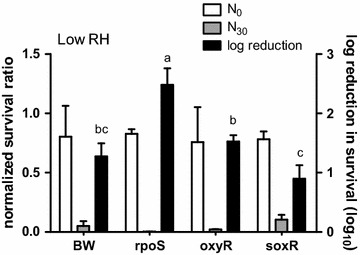
The normalized survival ratio and log reduction in survival of the bacteria at low RH. The normalized survival ratio before (N_0_) and after 30 min (N_30_) of aerosolization (*left Y-axis*) and log reduction in survival (*right Y-axis*) of the parental strain (BW), ∆*rpoS,* ∆*oxyR* and *∆soxR*. Temperature: 20 ± 2 °C. *Error bar*s represent the standard deviation of replicates (n = 3). The log reduction of different mutants was statistically analyzed by one-way ANOVA. Grouping was conducted with post-hos test Duncan analysis, and the *letters* above the *bars* represent different grouping

The process of aerosolizing *E. coli* from the current liquid medium into the air is detrimental, which causes a significant loss in bacterial viability. Although a high RH condition (Fig. 1) preserved the bacteria the most compared to the intermediate (Fig. 2) and low RH (Fig. 3), the log reduction in survival of the parental strain at high RH still reached to about 0.5 log (Fig. 1), which is equivalent to less than 32% of the bacteria that survive from the aerosolization process. At high RH, only ∆*rpoS* had a lower survival (1.5 log reduction) than the parental strain (0.5 log reduction), and both ∆*oxyR* and ∆*soxR* had the same survival as the parental strain. At intermediate RH, which the RH decreased from 90 to 40–60%, both ∆*rpoS* and ∆*oxyR* exhibited a higher log reduction in survival than the parental strain (Fig. 2). The log reduction of the parental strain and ∆*rpoS* was 0.7 log and 1.9 log, respectively. This means that approximately 94% less survival of ∆*rpoS* compares to the parental strain under this RH. For ∆*oxyR*, this mutant had a log reduction of 1.2 log, which means 68% less survival of this mutant than the parental strain. This result also demonstrates that missing the *rpoS* gene is more harmful to the bacteria than missing the *oxyR* gene. Again the log reduction of ∆*soxR* was similar to that of the parental strain at intermediate RH.

When the RH level was further adjusted to less than 40%, there was a significant loss in the bacterial viability across all the tested bacteria (Fig. 3). The log reduction of the parental strain went up to above 1.3 log i.e. only 5% of the bacteria from the liquid medium survived in the air. Interestingly, although the decrease in RH from an intermediate level (40–60%) to a low level (40–30%) was less than that from a high level (90%) to an intermediate level, the change in the viability of the parental strain was greater between intermediate and low RH than that between high and intermediate RH; log reduction at high RH—0.5 log, intermediate RH—0.7 log and low RH—1.3 log. The survival of airborne ∆*rpoS* was barely detectable at low RH. The log reduction of ∆*rpoS* at low RH was the highest among all the bacteria across all the RH conditions (about 2.5 log i.e. only 0.32% of survival after aerosolization). Although ∆*oxyR* was shown to be important for the bacterial survival at an intermediate RH, this mutant was no longer important at low RH as this mutant had the same viability as the parental strain. Finally, ∆*soxR* still showed no effect on the bacterial viability at low RH compared to the parental strain.

## Discussion

### General stress-*rpoS* gene

Most of the bioaerosol studies investigated the effect of RH on bacterial viability were conducted decades ago by measuring some morphological or physiological changes of the bacteria as the end points (Cox and Baldwin 1967; Hess 1965; Bateman et al. 1961; Dunklin and Puck 1948). Various inactivation mechanisms and molecular targets were suggested such as a reduction in RH inactivates bacteria by increasing their water loss; dehydration causes mechanical damage to the cell surface, ultimately killing the bacteria as well as a reduction in RH increases the oxygen diffusion into the bacteria and so increases the oxidative stress that could damage DNA and protein. This approach is mainly focused on the inactivation efficiency and bacterial damage as if all that bacteria are doing in the air is waiting for something to destroy them. No study has yet investigated whether bacterial stress response mechanisms have an impact on the survival of airborne bacteria. Recent physical modeling approaches revealed the potential physical change of the bioaerosols during droplet evaporation (Parienta et al. 2011). This result further hints the extensive environmental variations and challenges that the bacteria need to overcome in order to maintain their viability in the air such as against the increasing osmotic stress, cold stress and solute toxicity in the droplet nuclei. *rpoS* gene is one of the most well-studied and important master regulators of general stress-response genes. Various effector genes are directed by the *rpoS* regulator to initiate strategies to deal with stress, such as oxidative-stress response (*katE*, *katG*), osmoregulation (*otsA*, *osmY*), DNA protection (*dps)* and DNA repair (*recA*, *xthA*) (Battesti et al. 2011; Eisenstark et al. 1996). ∆*rpoS* is the only tested mutants showed a consistent higher log reduction than that of the parental strain at all RH conditions. This result indicates that *rpoS* plays a very important role in the survival of bioaerosols, and it is consistent with our observation that exponential phase *E. coli*, which the *rpoS* gene has not yet expressed, was susceptible to airborne suspension. In environmental and infection control applications, this result also implies that if the bacteria encounter other stresses that trigger the expression of the *rpoS* gene before aerosolization [e.g. some environmental pollution, resource limitation, drug treatments, and disinfectants are known to induce the general stress response (Battesti et al. 2011; Chung et al. 2006; Eisenstark et al. 1996)], this general stress response may also improve the airborne viability of the bacteria.

### Oxidative stress-*oxyR* and *soxR genes*

Hess (1965) showed that the increase in oxidative stress caused by low RH rather than dehydration alone was the primary cause of cell death. Cox and Baldwin (1967) also verified that the oxygen content in the air determined the survival of bacteria at a low RH (40%), but had no effect at a high RH (90%). Our results are consistent with their finding. The absence of the *oxyR* gene did not affect *E. coli* survival at a high RH (>90%) only at an intermediate RH (40–60%). As ∆*oxyR* is responsible for the oxidative stress due to elevated levels of H_2_O_2_ (Kullik et al. 1995), this result suggests that bacteria suffer from oxidative stress during airborne suspension at intermediate RH but the expression of *oxyR* gene reduces the impact of this stress. Since the log reduction of ∆*rpoS* was higher than that of ∆*oxyR* at an intermediate RH, other stress protected by the *rpoS* gene co-existed with this oxidative stress. The survival of the ∆*soxR* was the same as that of the parental strain at every RH level indicating that *soxR* did not play a role in defending *E. coli* against oxidative stress in the air. We expected that the log reduction of the ∆*oxyR* would be even more at RH below 40% than that at above 40% (i.e. assuming a higher oxidative stress level at a lower RH) but it was not the case; the log reduction in survival of the ∆*oxyR* was the same as that of the parental strain at this RH. This result may imply that other types of stress more detrimental than H_2_O_2_ oxidative stress were produced when RH fell below 40%. This postulation is supported by our finding that the ∆*rpoS* had the highest log reduction at <40% RH. The stress exerted at <40% RH was severe and required a response related to the *rpoS* gene rather than the *oxyR* gene to maintain the bacterial viability. By using specific mutants that have a known stress-response gene deleted, we could better study the nature of the stress under different RH. For instance, this study discovered that the damage caused by the oxidative stress produced in the atmosphere could be reduced by *oxyR* gene, which responds to elevated levels of H_2_O_2_ in the bacteria (a more specific understanding of oxidative stress compared to previous studies). Several genes under the regulation of rpoS are responsible for oxidative stress response e.g. *katE* and *katG* (Battesti et al. 2011; Eisenstark et al. 1996). Double mutations of *rpoS* and *oxyR* may further reduce the bacterial survival. As shown in previous bioaerosol studies that temperature and RH conditions affect the survival of airborne bacteria (Tang 2009; Mohr 2007). Some researchers have proposed that adjusting indoor temperature and RH may reduce the viability of airborne bacteria as a low-cost infection control technology in health-care environments (Tang 2009; Mohr 2007). This study contributes to some new knowledge to advance our understanding of the stress and stress-response genes relevant to different RH conditions that may help developing this infection control technology in the future.

### Implications of the study

This study is the first to prove the concept that stress-response genes are also vital for bacterial survival in the air. Bacteria are subjected to *oxyR*-associated oxidative stress at intermediate RH and *rpoS*-associated general stress at all RH in the atmosphere. This study is important to verify and explain some of the observations reported in previous bioaerosol studies and demonstrates a new approach to explore the biological mechanisms associated with the viability of airborne bacteria. With the support from this study, more experiments can be designed to investigate the effect of bacterial solution on the stress response of different airborne bacteria. For instance, human body fluids contain proteins and many other biological solutes, which are much more complicated than PBS alone. Different solutions may create a different type and intensity of stress to the bacteria and/or affect the bacterial response to the same stress. Similarly, we used *E. coli* as a model in this study but future studies can look at airborne bacterial pathogens and bacteria that are relevant to various environmental processes.

## Additional file

**Additional file 1.** Additional information.

## Abbreviations

*E. coli*: *Escherichia coli*
RH: relative humidity
PBS: phosphate buffer saline
TSA: tryptone soy agar
qPCR: quantitative polymerase chain reaction
ANOVA: one-way analysis of variance
