# Supplementary material for: Importance of stress-response genes to the survival of airborne Escherichia coli under different levels of relative humidity
Source: AMB Express. 2017 Mar 24;7:71. doi: 10.1186/s13568-017-0376-3 (PMC5366994; doi:10.1186/s13568-017-0376-3)
Supplement: Supplementary file 1 — Additional file 1. Additional information. [file 13568_2017_376_MOESM1_ESM.docx]

**Journal name: Applied Microbiology and Biotechnology Express**

**Manuscript title: Importance of stress-response genes to the survival of airborne *Escherichia coli* under different levels of relative humidity**

Authors: Tsz Wai NG, Wing Lam CHAN, Ka Man LAI^*^

Affiliation and address of the authors: Department of Biology, Hong Kong Baptist University, Kowloon Tong, Hong Kong Special Administrative Region.

**______________________________________________________**

^*^Corresponding authors. Tel.: +852 3411 5835; e-mail: [laikaman@hkbu.edu.hk](mailto:laikaman@hkbu.edu.hk).

**Effect of nebulization and filter sampling on bacterial survival**

Methods:

The survival percentage of the bacteria was determined before and after nebulization by using plate-counting method to prove that nebulization did not inactivate the bacteria. An aliquot of the bacterial solution was taken out from the nebulizer. After appropriate dilution, the diluents were plated on tryptone soy agar (TSA) and incubated at 37 ^o^C for 24 h before counting the colony forming units.

Bacterial survival on filter under different filtration time was investigated. Two filters were used simultaneously to collect the airborne bacteria after 30 min of suspension. After 3 min of filter sampling, one of the filters was removed (3 min filtering), while another filter was kept running for another 3 min in blank air without bacteria under the same temperature and RH conditions (6 min filtering). Then the log reduction of the bacteria between these two filters was compared. It is imperative that adequate bacteria is collected for analysis, and 3 min sampling time was determined to be the appropriate period in our preliminary experiments. The bacteria on the filter were challenged for an extra 3 min of filtration because it is assumed that the potential inactivation effect due to filter sampling follows the exponential decay pattern (i.e. decay rate is constant over time).

In another experiment, after nebulization, bacterial solution with the parental strain or ∆*rpoS* mutant was spiked directly onto the same type of filter materials used in air sampling and filtered. Filter-sterilized room air at 60% RH and 20 ^o^C passed through the filter at a flow rate of 28 L/ min, the same flow rate as in air sampling for 20, 40 and 60 min. The bacterial counts on the filter at time 0 min and other filtration times were determined by eluting the bacteria from the filter into phosphate buffer saline (PBS) and then plating on TSA. The bacterial colonies were counted after 24 h of incubation at 37 ^o^C.

Results

Effect of nebulization on bacterial survival

Fig. S1. Survival percentage of the parental strain (BW) and different *E. coli* mutants after nebulization for 3 min. Nebulization pressure: 20 psi at room temperature (20±2 ^o^C). Error bars represent the standard deviation of replicates (n=3). The survival percentage of the bacteria was statistically analyzed by one-way ANOVA and no significant difference was detected.

Effect of prolonged filtration on the survival of airborne bacteria deposited on the filter

Fig. S2. Log reduction in bacterial survival at 3 and 6 min filtering time. Temperature = 20^o^C; ∆*soxR* was not included in the experiment because this mutant had the same log reduction as the parental strain (BW). ∆*oxyR* was not included in the experiments at high and low RH because this mutant had the same log reduction as the parental strain (BW) at these two levels of RH. Error bars represent the standard deviation of replicates (n=3). The log reduction of different bacteria was statistically analyzed by one-way ANOVA. Grouping was conducted with post-hos test Duncan analysis, and the letters above the bars represent different grouping.

Effect of filtration on the survival of bacteria spiked on filter surface

Fig. S3. Effect of filtration on the survival of bacteria spiked on filter surface. Room air at 60% RH and 20 ^o^C was filter-sterilized and passed through the filter at a flow rate of 28 L/ min.

**Statistical analysis**

Methods:

All the log reduction in bacterial survival under different RHs was compared in a single model using one-way analysis of variance (ANOVA) with Duncan’s post-hoc test (SPSS v. 23). The difference between means with a *p*-value lower than 0.05 (*p* < 0.05) was regarded as statistically significant.

Results:

Fig. S4. The normalized survival ratio and log reduction in survival of the bacteria at high RH. The normalized survival ratio before (N_0_) and after 30 min (N_30_) of aerosolization (left Y-axis) and log reduction in survival (right Y-axis) of the parental strain (BW), ∆*rpoS,* ∆*oxyR* and *∆soxR*. Temperature: 20±2 ^o^C. Error bars represent the standard deviation of replicates (n=3).

Fig. S5. The normalized survival ratio and log reduction in survival of the bacteria at intermediate RH. The normalized survival ratio before (N_0_) and after 30 min (N_30_) of aerosolization (left Y-axis) and log reduction in survival (right Y-axis) of the parental strain (BW), ∆*rpoS,* ∆*oxyR* and *∆soxR*. Temperature: 20±2 ^o^C. Error bars represent the standard deviation of replicates (n=3).

Fig. S6. The normalized survival ratio and log reduction in survival of the bacteria at low RH. The normalized survival ratio before (N_0_) and after 30 min (N_30_) of aerosolization (left Y-axis) and log reduction in survival (right Y-axis) of the parental strain (BW), ∆*rpoS,* ∆*oxyR* and *∆soxR*. Temperature: 20±2 ^o^C. Error bars represent the standard deviation of replicates (n=3).

In summary, this grouped data model gave a slightly different result from the separated RH models, in which the ∆*oxyR* had a higher log reduction than the parental strain at both intermediate and high RH in the grouped model (Fig. S4 & 5). This different result is because of the statistical comparison of the data with a much greater range and variation across all the tested RH conditions and mutants.
